# NLRC5 Serves as a Pro-viral Factor During Influenza Virus Infection in Chicken Macrophages

**DOI:** 10.3389/fcimb.2020.00230

**Published:** 2020-05-19

**Authors:** Shubhada K. Chothe, Ruth H. Nissly, Levina Lim, Gitanjali Bhushan, Ian Bird, Jessica Radzio-Basu, Bhushan M. Jayarao, Suresh V. Kuchipudi

**Affiliations:** ^1^Penn State Animal Diagnostic Laboratory, Department of Veterinary and Biomedical Sciences, Pennsylvania State University, University Park, PA, United States; ^2^Applied Biological and Biosecurity Research Laboratory, Pennsylvania State University, University Park, PA, United States

**Keywords:** NLRC5, chicken, avian influenza, NFκB, macrophage

## Abstract

Avian influenza viruses (AIVs) cause major economic losses to the global poultry industry. Many host factors have been identified that act as regulators of the inflammatory response and virus replication in influenza A virus (IAV) infected cells including nucleotide-binding oligomerization domain (NOD) like receptor (NLR) family proteins. Evidence is emerging that NLRC5, the largest NLR member, is a regulator of host immune responses against invading pathogens including viruses; however, its role in the avian immune system and AIV pathogenesis has not been fully explored. In this study, we found that NLRC5 is activated by a range of low and highly pathogenic AIVs in primary chicken lung cells and a chicken macrophage cell line. Further, siRNA mediated NLRC5 knockdown in chicken macrophages resulted in a significant reduction in AIV replication which was associated with the upregulation of genes associated with activated NFκB signaling pathway. The knockdown of NLRC5 enhanced the expression of genes known to be associated with viral defense and decreased innate cytokine gene expression following AIV infection. Overall, our investigation strongly suggests that NLRC5 is a pro-viral factor during IAV infection in chicken and may contribute to pathogenesis through innate cytokine regulation. Further studies are warranted to investigate the IAV protein(s) that may regulate activation of NLRC5.

## Introduction

Influenza A viruses (IAVs) continue to spread globally and have increased the potential to cause epidemics due to their wide host range and mutation frequency. Avian influenza viruses (AIVs), have caused a significant socio-economic impact on global poultry production, trade and public health (Capua et al., 2002; Alexander, 2007; Basuno et al., 2010). While ducks and waterfowl are considered natural reservoirs for IAVs and typically harbor asymptomatic infection, the disease in chickens can range from mild to severe or even fatal in the case of highly pathogenic avian influenza viruses (HPAIVs). In such cases, death typically occurs within 6 days, before an adaptive immune response, suggesting that innate immune mechanisms play a crucial role. While the underlying molecular mechanisms that lead to this disparate pathogenesis are not fully understood, it has been suggested that the elevation of cytokines during HPAIV infection contributes to the mortality (Karpala et al., 2011; Moulin et al., 2011) and that rapid cell death could have a protective role (Kuchipudi et al., 2012).

Nucleotide-binding oligomerization domain-containing (NOD)-like receptors (NLRs) have gained increased attention in recent years for their role in the modulation of innate immune responses, modulating a wide array of pathways in response to viral infection. The CARD domain containing 5 (NLRC5) protein is the largest NLR member and is highly expressed in the myeloid and lymphoid immune cell lineages (Kuenzel et al., 2010). While NLRC5 has been well-described as an inducer of MHC I genes in many cell types (Kobayashi and Van Den Elsen, 2012), various roles have been demonstrated in recent years, sometimes showing contradicting effects to innate immune processes. For example, the siRNA-mediated knockdown of NLRC5 expression in various cell types has shown both enhancement (Cui et al., 2010) as well as the decrease of interferon (IFN) response (Kuenzel et al., 2010), suggesting NLRC5 can act as a positive and negative regulator of IFN response during viral infection. These contradictory reports also suggest that the role of NLRC5 could be cell type and/or stimulus specific (Ranjan et al., 2015).

Although a few other members of the NLR family have been studied for their differential regulation during IAV infection, such as NLRP3, NRLX1, and NOD 2 (Allen et al., 2009; Kanneganti, 2010), limited work has been done to evaluate the role of NLRC5. Likewise, the function of the NLR family members has not been well-characterized in avian species. The one study performed to date demonstrated the upregulation of NLRC5 within 2 h of lipopolysaccharide treatment, suggesting it plays a role early in the immune response (Lian et al., 2012). Therefore, in this study, we have investigated the regulation of NLRC5 in chicken cells infected with both low and high pathogenicity AIVs. Further, we have investigated the role of NLRC5 in the modulation of antiviral cytokine response and demonstrated that NLRC5 plays a role in regulating the replication of AIV in chicken macrophages.

## Materials and Methods

### Cell and Virus Propagation

**Figure 1:**
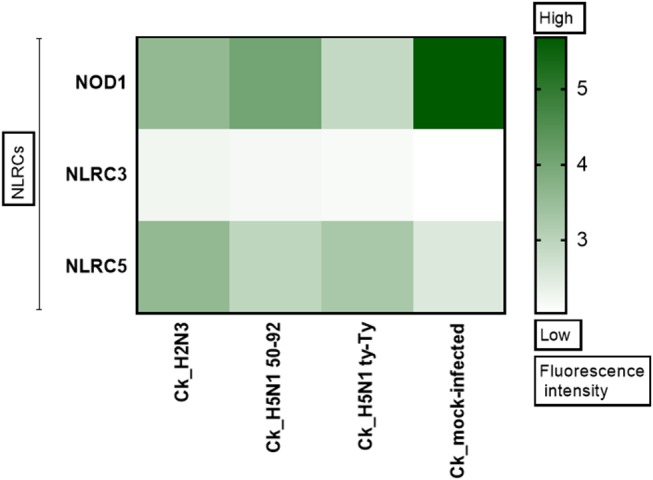
NLRC5 expression is upregulated after AIV infection. The heat map shows the expression of three Nod Like Receptor (NLR) genes in primary chicken lung cells at 24 h after infection with LPAIV A/mallard/duck/England/7277/06 (LPAI-H2N3), classical lineage HPAIV A/turkey/England/50-92/91 (H5N1-tyEng91) or contemporary Eurasian lineage HPAIV A/turkey/Turkey/1/05 (H5N1-tyTR05). Color corresponds to the expression levels of each gene from 0 (low expression) to >5 (high expression).

With the exception of the initial transcriptome analyses (Figure 1) and chicken embryo fibroblast (CEF) cells infection assay (Figure S1), all assays were performed in chicken macrophage MQ-NCSU cells. The MQ-NCSU and CEF cells were grown at 37°C with 5% CO_2_ in Dulbecco's modified Eagle's medium (DMEM), 10% fetal bovine serum (FBS), and 1% PSA (100 units/mL of penicillin, 100 μg/mL of streptomycin, and 0.25 μg/mL of Amphotericin B), all from Corning (Corning, NY, USA).

Viruses used in this study include: Low pathogenic avian influenza viruses (LPAIVs) A/mallard/duck/England/7277/06 (H2N3), A/chicken/Pennsylvania/7659/1985 (H5N2) and A/chicken/Pennsylvania/3779-2/1997 (H7N2) and highly pathogenic avian influenza viruses (HPAIVs) A/turkey/England/50-92/91 (H5N1) of classical lineage, A/turkey/Turkey/1/05 (H5N1) of contemporary Eurasian lineage, and A/Vietnam/1203/2004 (H5N1). All viruses were propagated in 9- to 11-day old embryonated chicken eggs as described elsewhere (Brauer and Chen, 2015). Briefly, the chicken eggs were candled to aid in marking the air sac and relatively low vascular region. The eggshell was pierced at the marked location and 100 μl of virus inoculum was delivered to the allantoic cavity using a tuberculin syringe with a 26-gauge needle. The eggshell was sealed with glue, and the eggs were placed in an egg incubator at 37°C. At 72 h post infection (hpi), the allantoic fluid was harvested and centrifuged at 330 g for 15 min. The supernatant was aliquoted and stored at −80°C. All experiments involving HPAIV were performed under biosafety level-3 (BSL-3) conditions. All LPAIV experimentation was performed in BSL-2.

### Transcriptome Analysis in Primary Chicken Lung Cells

Primary cell cultures were prepared in house using lungs of 4 wk old broiler chickens. Fine lung pieces were trypsin-digested overnight at 4°C. Large undigested tissue pieces were removed using a cell strainer and the remaining suspension was centrifuged at 1,200 × g for 5 min. The resultant cell pellet was reconstituted in small volume of cell culture medium. Primary chicken lung cells were infected with A/mallard/duck/England/7277/06 (H2N3) LPAIV, A/turkey/England/50-92/91 (H5N1) HPAIV, or A/turkey/Turkey/1/05 (H5N1) HPAIV. Mock infections were performed using phosphate buffered saline (PBS). Total RNA was collected at 24 hpi, and microarray analysis was performed to evaluate the global gene expression as previously described (Kuchipudi et al., 2014). Microarray datasets are available on the gene expression omnibus (GEO) site under accession number *GSE33389*. Gene expression analysis was carried out using R build 3.3.1 [Bioconductor (V3.4)]. The core Bioconductor algorithms, including necessary library packages essential for the analysis of raw transcriptomic data, were installed locally before execution. The data analytical command line scripts were run through R studio (1.1.383), an integrated development environment interface for R. The Robust Multichip Averaging probe summarization algorithm described previously (Irizarry et al., 2003a,b,c) allowed for background correction and normalization of the data. Comprehensive annotation of normalized data yielded log expression values of over 33,000 transcripts, which were transferred to a spreadsheet for further grouping and selection of genes. Expression log values (duplicates) of pre-identified genes (selected based on peer-reviewed publications (Hers et al., 2011; Ehrlich et al., 2017) as well as commercial assay kits) were then plotted in Prism 7 (GraphPad; San Diego, CA, USA) for visual interpretation.

### Transcript Quantification

For the quantification of host transcripts, total RNA was extracted from cells using RNeasy Plus Mini Kit following the manufacturer's instructions (Qiagen, Germantown, MD, USA), then quantified using NanoDrop™ Lite Spectrophotometer (Thermo Fisher Scientific, Waltham, MA, USA). The cDNA was prepared with the qScript cDNA Synthesis Kit (Quantabio, Gaithersburg, MD, USA) using 1 μg of the total RNA per sample. The quantitative PCR for host gene amplification was performed with the Power SYBR Green Master Mix (Thermo Fisher Scientific) using a 1:10 dilution of cDNA and the primer sets shown in Table 1. The PCR reaction was run at 95°C for 10 min followed by 40 cycles of 95°C for 15 s and 60°C for 60 s. Melt curve analysis was performed for each PCR run to ensure a single peak indicating a single amplicon resulting from the reaction. Hydroxymethylbilane synthase (HMBS) was used as the house keeping gene for relative quantification of gene expression using the ΔΔCt method described previously (Livak and Schmittgen, 2001). Gene expression levels are expressed as fold-change compared with HMBS expression.

**Table 1:** List of primers used in the study.

	**Forward**	**Reverse**	**Genbank ID**	**References**
**NLRC5**	TGAGCTACACGTCAGGAAGGA	GCTCTGCAGAATGGACACAA	NM_001318435.1	Lian et al., 2012
**NLRP3**	GGTTTACCAGGGGAAATGAGG	TTGTGCTTCCAGATGCCGT	KJ470775.1	Ye et al., 2015
**IL-1β**	TGCTGGTTTCCATCTCGTATGT	CCCAGAGCGGCTATTCCA	XM_015297469.1	Chhabra et al., 2016
**IL18**	AGGTGAAATCTGGCAGTGGAAT	ACCTGGACGCTGAATGCAA	AJ276026	Suzuki et al., 2009
**HMBS**	GGCTGGGAGAATCGCATAGG	TCCTGCAGGGCAGATACCAT	XM_417846.2	Samiullah et al., 2017
**Matrix**	AGATGAGTCTTCTAACCGAGGTCG	TGCAAAAACATCTTCAAGTCTCTG	MN400394.1	Spackman et al., 2002

Viral RNA was extracted from infected cell culture supernatants using the QIAamp Viral RNA Mini Kit (Qiagen). The viral matrix (M) gene was amplified with the SuperScript™ III Platinum™ One-Step qRT-PCR Kit (Invitrogen, Carlsbad, CA, USA) using primers and Taqman probe previously described (Spackman et al., 2002).

### Virus Titer Determination

Calculation of the relative equivalent units (REUs) was based on influenza viral M gene amplification. For REU calculation, viral RNA was extracted from the supernatant of infected cells of known TCID_50_, serially diluted, and assayed by the viral M gene qRT-PCR. A standard curve of was plotted between TCID_50_ units and corresponding mean Ct values, and experimental viral RNA output Ct values were fitted to the standard curve to estimate REU TCID_50_ of each experimental sample.

The REU viral titers were calculated for cells infected with LPAIV H5N2 and H7N2 whereas the relative matrix gene quantification was carried out for cells infected with HPAIV H5N1 to reduce handling of the virus in BSL3 conditions. Both REU and relative matrix gene quantification are based on viral M gene amplification and provide equivalent information.

### IAV Infection Assay

All infection assays were performed in 6-well plates at a seeding density of 10^6^ cells/well and a multiplicity of infection (MOI) of 1. Cells were grown to 80% confluence and washed with PBS. Serum-free medium (UltraCULTURE™; Lonza, Walkersville, MD, USA) supplemented with 1 μg/ml of TPCK-trypsin and containing the desired amount of IAV was added to each well and pre-incubated for 2 h at 37°C and 5% CO_2_. The cells were then rinsed with PBS, and fresh supplemented media was added. Cells and supernatant were harvested at various time intervals and used in the previously described assays for virus quantification and relative host gene quantification, respectively. Three biological replicates were performed for each condition. The time course infections (4, 8, 12, 24, 48 hpi) were performed with LPAIV H5N2. The 24 hpi timepoint showed the most significant difference in NLRC5 mRNA expression. The future experiment with LPAIV H7N2 were carried out at 24 hpi. The HPAIV H5N1 experiments were performed in BSL3 laboratory to obtain the data for 24 hpi timepoint and a “late” 48 hpi timepoint as well.

### NLRC5 Protein Detection and Quantification

Immunofluorescence staining was used to visualize the NLRC5 protein in infected cells at 24 hpi. Cells grown on Nunc Lab-Tek four-chamber slides (Thermo Fisher Scientific), were fixed using acetone:methanol (1:1) for 10 min then incubated with primary anti-NLRC5 rat monoclonal (catalog MABF260 clone 3H8, EMD Millipore, Burlington, MA) at a concentration of 1:1000 in Tris-buffered saline followed with three washes with Tris-buffered saline washes. The cells were further incubated with goat polyclonal FITC-conjugated anti-rat IgG (catalog ab6840, Abcam, Cambridge, MA, USA) at a concentration of 1:500 for 30 min. The cells were mounted using ProLong Gold Antifade Mountant with DAPI (Invitrogen) and left at room temperature overnight in the dark for curing. The slides were observed under confocal microscope for imaging using an Fluoview 1000 (Olympus, Waltham, MA, USA). In addition, to quantify the expression of NLRC5, a quantitative sandwich ELISA was performed using a commercial ELISA kit (MyBioSource, San Diego, CA, USA) as per the manufacturer's instructions.

### siRNA-Mediated RNA Interference

The chicken macrophage MQ-NCSU cells were grown to 80% confluence in 6 well plates. A previously described NLRC5 gene specific siRNA sequence, 5′-CAUAACACUGCAGUCCUGAGGUUUA-3′ was synthesized (Life Technologies Corporation) to mediate the interference (Lian et al., 2012). Stealth RNAi™ siRNA Negative Control Med GC Duplex #3 (Invitrogen) was used as a negative scramble RNA control. Cells were transfected following the manufacturer's instructions using Lipofectamine™ RNAiMAX (Invitrogen). Thirty-two hours post-transfection, the cells were infected with AIV at MOI 1. Three biological replicates were used in each treatment. Cells were harvested at 32 h post transfection (hpt) and 8, 24, and 48 hpi, and the supernatant was analyzed for host gene expression and virus quantification.

### RT^2^ Profiler PCR Array and Analysis

The RT^2^ Profiler™ PCR Array Chicken Innate & Adaptive Immune Responses (Qiagen) is a SYBR Green qPCR-based gene expression analysis system. Cells were infected with LPAIV H5N2 and total RNA was extracted at 24 hpi. RT^2^ First Strand Kit (Qiagen) was used to generate cDNA, and cDNA was used in the PCR Array using RT^2^ SYBR Green ROX qPCR Mastermix following the manufacturer's instructions. Data was analyzed for fold-change determination using Qiagen's GeneGlobe data analysis center web resource (https://www.qiagen.com/us/shop/genes-and-pathways/data-analysis-center-overview-page/). The significance of the change in gene expression between the two samples was evaluated by unpaired Student *t*-test for each gene by the software. The level of statistical significance was set at < 0.005.

RT^2^ Profiler Array data was further subjected to pathway analysis using Qiagen's Ingenuity Pathway Analysis (IPA) software to analyze gene expression data, identify upstream regulators and plot the differentially expressed genes in relation to the signaling pathways. The RT^2^ profiler PCR expression fold change data for the experimental and control samples was utilized in the IPA analysis to determine the most significantly affected pathways.

### Statistical Analysis

Results of mock-infected and virus-infected treatment conditions were compared by unpaired two-tailed t- test (GraphPad Prism v7) with Welch's correction and 95% confidence interval. Differences were considered significant when the *p* < 0.05.

## Results

### NLRC5 Expression Is Upregulated During Both LPAIV and HPAIV Infection

NLRs play a key role in immune responses, however their role during IAV infection in the chicken remains unexplored. To determine if members of this family are upregulated during IAV infection, microarray analysis was performed in primary chicken lung cells after infection with classical or Eurasian lineage H5N1 HPAIVs, H2N3 LPAIV, or sterile PBS. NLRC5 was observed to be upregulated in each of the AIV infections compared with the PBS mock infection control (Figure 1).

**Figure 2:**
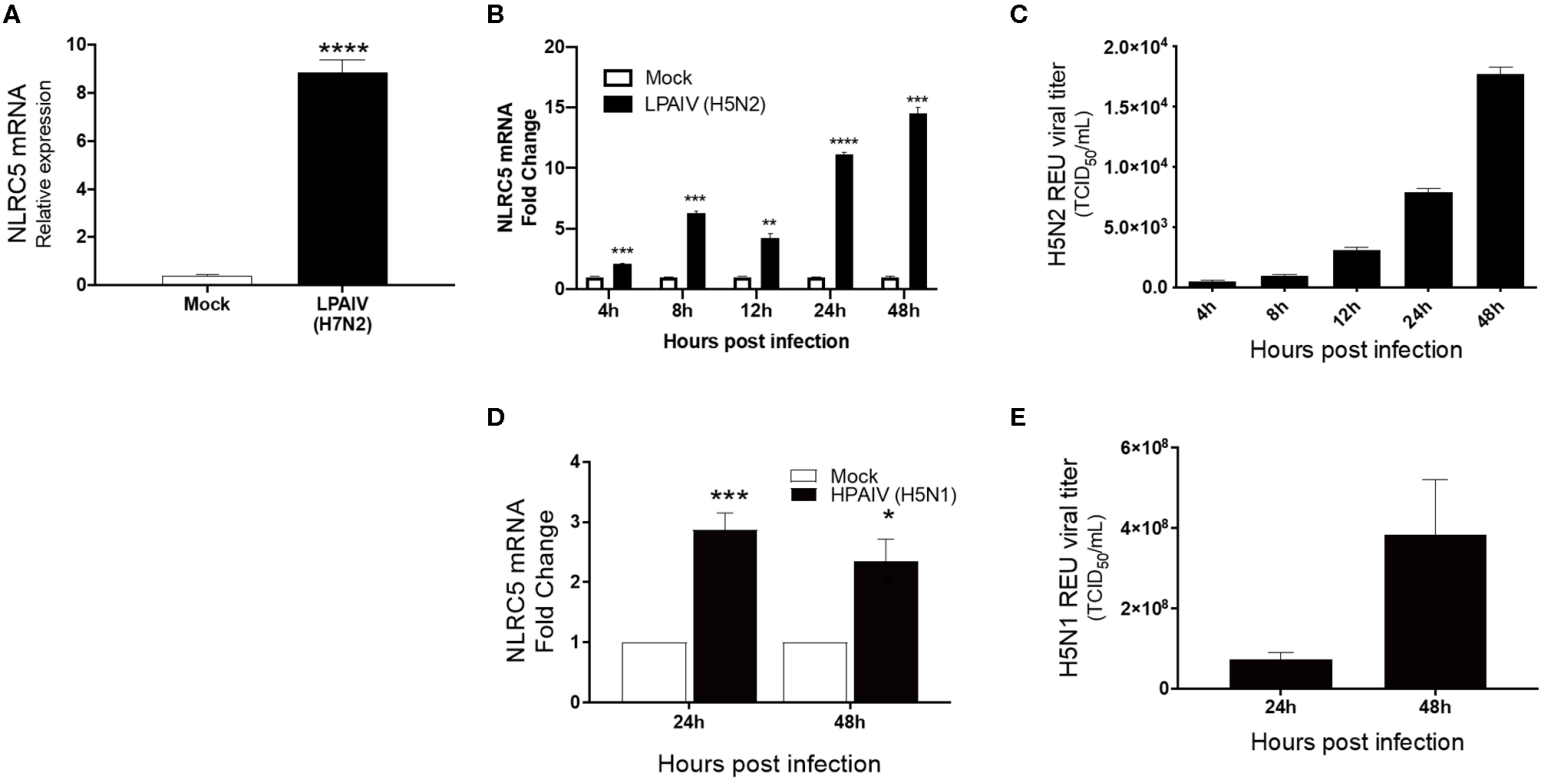
Increase in NLRC5 mRNA and virus production in HPAIV- and LPAIV-infected chicken macrophages. Chicken macrophage MQ-NCSU cells were infected with the LPAIV H7N2, H5N2, or HPAIV H5N1. Total cellular mRNA and viral supernatant were collected at the designated timepoints. **(A)** NLRC5 mRNA levels at 24 hpi with H7N2 are shown. After LPAIV H5N2 infection, **(B)** NLRC5 mRNA levels and **(C)** virus production in the supernatant were measured between 4 and 48 hpi. After HPAIV H5N1 infection, **(D)** NLRC5 mRNA levels and **(E)** virus production in the supernatant were measured at 24 and 48 hpi. All NLRC5 mRNA levels were normalized to the housekeeping gene, HMBS. Data represents average of three biological replicates with error bars showing standard deviation. ^*^p ≤ 0.05, ^**^p ≤ 0.01, ^***^p ≤ 0.001, ^****^p ≤ 0.0001.

Macrophages act as the first line of defense following infection and are known to be susceptible to infection by both LPAIVs and HPAIVs. Besides, excessive pro-inflammatory cytokine production is a major pathogenicity factor during HPAIV infection in chicken, and macrophages are the major contributors of cytokines during viral infection (Kuribayashi et al., 2013). To determine whether IAV infection influences the regulation of NLRC5, we used chicken macrophage MQ-NCSU cells for these studies. MQ-NCSU cells were infected with three different subtypes: LPAIV H7N2, LPAIV H5N2, or HPAIV H5N1. In both LPAIV infections, NLRC5 was upregulated. At 24 hpi, infection with LPAIV H7N2 caused a significant increase in NLRC5 mRNA expression relative to the housekeeping gene HMBS from 0.40 ± 0.057 times in mock-infected cells to 8.86 ±0.499 times in LPAIV-infected cells (Figure 2A). In the case of LPAIV H5N2, not only was a similar increase noted, but the expression increased over a time course of 2 days (Figure 2B). We also saw an increase in viral titer over the same time period (Figure 2C) which was positively correlated with the increase in NLRC5 transcript (Pearson's *r* = 0.9515, *p* = 0.0127, two-tailed). This increase in NLRC5 mRNA was also seen in CEF cells infected with H5N2, albeit lower than observed in the macrophages (Figure S1). Infection with the HPAIV also resulted in an increase in NLRC5 mRNA (Figure 2D) as well as an increase in viral titer (Figure 2E).

**Figure 3:**
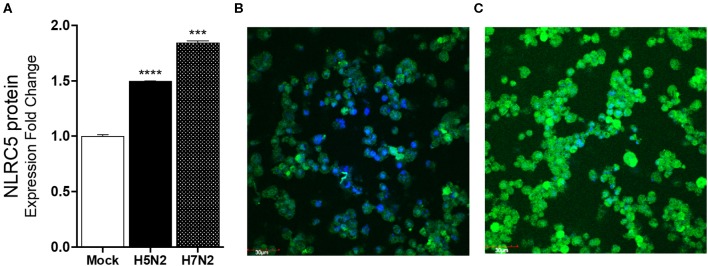
NLRC5 protein is increased in LPAIV-infected chicken macrophages. Chicken macrophage MQ-NCSU cells were infected with the H7N2 or H5N2 AIVs for 24 h. **(A)** NLRC5 protein was quantified using ELISA. **(B)** Fluorescence photomicrographs of **(B)** mock-infected cells stained for NLRC5 and **(C)** LPAIV H5N2-infected macrophage cells. The blue color represents DAPI-nuclei specific staining and the green color represents FITC-NLRC5 specific staining. Data points are mean of three biological replicates with error bars showing standard deviation. ^***^p ≤ 0.001, ^****^p ≤ 0.0001.

To confirm that the increasing mRNA levels reflect an increase in protein, ELISA and immunostaining for NLRC5 were performed. Consistent with the mRNA levels, greater levels of NLRC5 protein was found in LPAIV H5N2 infected cells as compared to mock-infected cells at 24 hpi using ELISA (Figure 3A). Relative protein expression in cells infected with the H5N2 and the H7N2 caused a 1.5- and 1.8-fold increase in measured NLRC5 protein as compared to mock infection, respectively. This increase was further confirmed by immunofluorescence (Figures 3B,C). Taken together, microarray, qPCR and protein assay results show that NLRC5 gene expression is increased in chicken macrophages during both LPAIV and HPAIV infection in a subtype-independent manner.

### Decreased Expression of NLRC5 Results in Decreased IAV Replication in Chicken Macrophages

**Figure 4:**
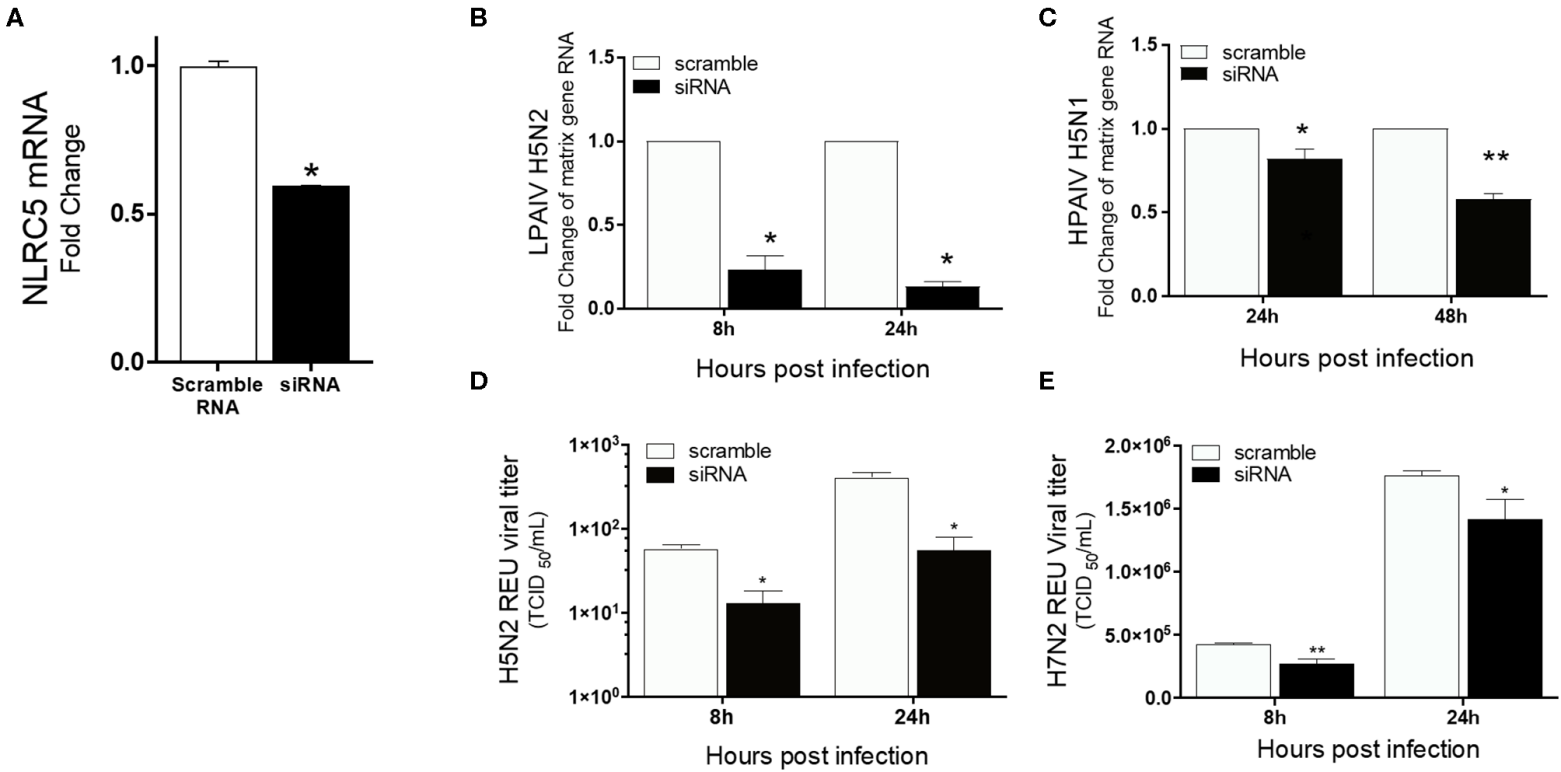
Reduced virus titers of LPAIV and HPAIV in NLRC5 KD chicken macrophages. MQ-NCSU cells were transfected with NLRC5 siRNA or non-specific control siRNA (scramble) and infected with H5N2, H7N2 or H5N1 at 32 hpt. NLRC5 mRNA and viral RNA were quantified by qRT-PCR. **(A)** NLRC5 mRNA levels were measured at 32 hpt and compared to control. Viral RNA levels were measured by M-gene amplification at 8 and 24 hpi with **(B)** H5N2 and **(C)** H5N1. Supernatant infectious virus production was measured using relative equivalence units (REUs) at 8 and 24 hpi with **(D)** H5N2 or **(E)** H7N2. Data represents average of three biological replicates with error bars showing standard deviation. ^*^p ≤ 0.05, ^**^p ≤ 0.01.

To confirm the role of NLRC5 in AIV replication, siRNA mediated NLRC5 knockdown (KD) was carried out in chicken macrophages. At 32 hpt, the NLRC5 levels were found to be approximately 50% lower in siRNA-treated cells as compared to the control transfection (Figure 4A). The KD cells were next infected with the H5N2 LPAIV, H5N1 HPAIV or H7N2 LPAIV to observe the effect on virus production. NLRC5 KD cells showed significant reduction in virus replication compared to the wild type (scramble control) cells in both the H5N2 and H5N1 infections albeit to a greater level in the low pathogenicity H5N2 infection (Figures 4B,C). Likewise, the corresponding viral titers in supernatants from KD cells infected with H5N2 and H7N2 were also decreased (Figures 4D,E), confirming the role of NLRC5 in AIV replication and suggesting that NLRC5 could be a pro-viral host factor.

### Decreased Expression of NLRC5 in AIV-Infected Macrophages Enhances Viral Defense and Decreases Innate Cytokine Production

**Figure 5:**
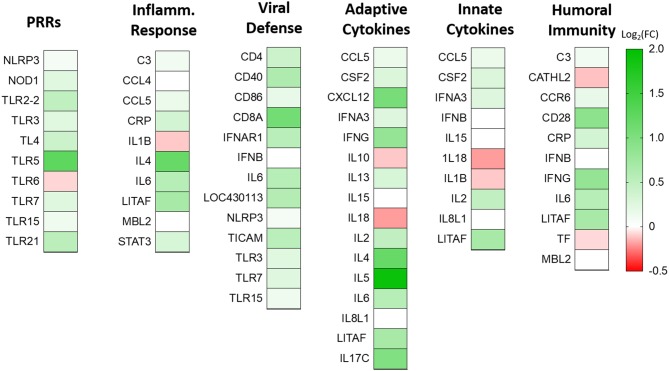
Effect of NLRC5 KD on differentially expressed immune genes during H5N2 infection. NLRC5 siRNA and scramble control transfected MQ-NCSU cells were infected with H5N2 virus. Total RNA was collected at 24 hpi and assayed by the RT^2^ Profiler PCR Array system. Selected genes taking part in innate and adaptive immune response are shown as log_2_ transform of fold-change expression relative to the gene expression in the mock-transfected cells. Data represent an average of three biological replicates.

**Figure 6:**
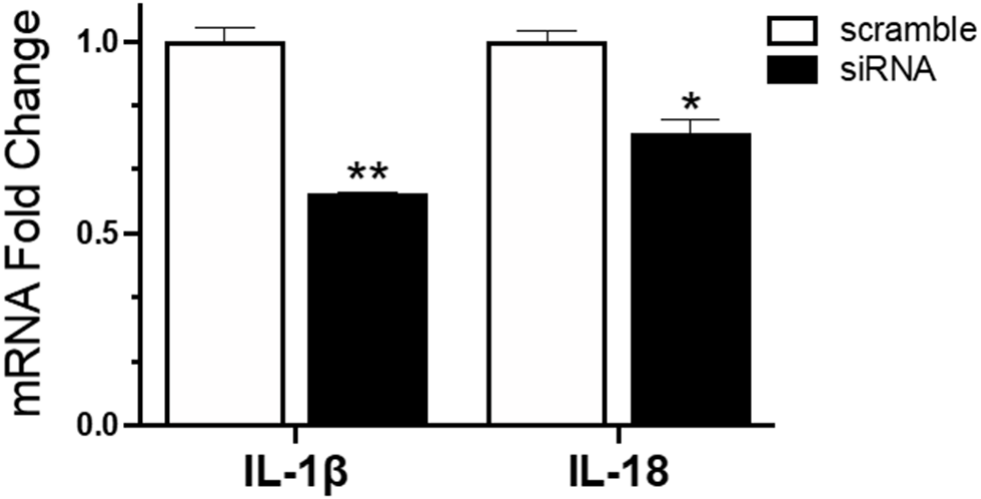
Reduced mRNA expression of IL-1β and IL-18 in IAV infected NLRC5 KD cells. MQ-NCSU cells were transfected with NLRC5 siRNA or non-specific control siRNA (scramble). The relative mRNA expression of IL-1β and IL-18 was determined at 32 hpt by quantitative RT-PCR and normalized to the housekeeping gene HMBS. Data represents average of three biological replicates with error bars showing standard deviation. ^*^p ≤ 0.05, ^**^p ≤ 0.01.

To understand the role of NLRC5 in the innate immune response to AIV in chicken macrophages, we compared the transcriptional profiles of NLRC5 KD and mock-transfected cells infected with LPAIV H5N2 using a chicken RT^2^ Profiler PCR array which allows simultaneous screening of expression levels of 84 pre-selected immune genes. From the full data set (Figure S2), variation in key functional groups was observed (Figure 5). Overall, pattern recognition receptor genes, genes relating to the inflammatory response, and genes involved in viral defense showed increased expression in the AIV-infected NLRC5 KD cells compared to control cells, suggesting that NLRC5 plays a role in the regulation of genes in these categories. Conversely, expression of innate cytokines such as IFN-β, IL-15, IL-18, IL-1β, and IL-8, were decreased. This result was further confirmed by evaluating the mRNA expression of two innate cytokines in the NLRC5 KD cells. Both IL-1β and IL-18 transcripts were lower in the NLRC5 KD cells as compared to the mock-transfected controls (Figure 6). Together, this data suggests that NLRC5 serves as an enhancer of innate cytokine production during AIV infection in chicken macrophages.

### IPA Analysis Suggests that NLRC5 Contributes to NFκB Regulation in AIV-infected Macrophages

**Figure 7:**
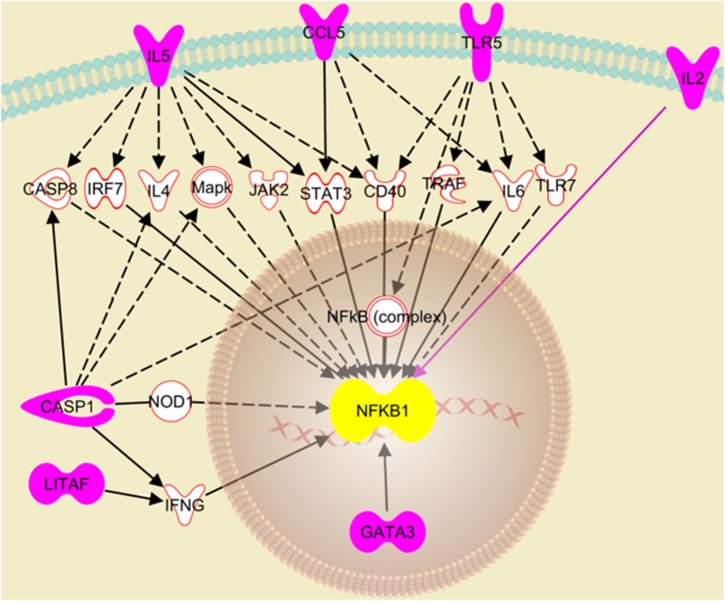
Putative pathway showing regulatory relationships of key genes to NFκB activation transcriptionally regulated by NLRC5. Ingenuity Pathway Analysis revealed seven genes (pink fill) which were significantly upregulated after NLRC5 KD macrophages were infected with LPAIV H5N2 and are indicative of activation of the NFκB gene response. The dashed lines represent an indirect relationship, and a solid black or pink line represents a direct relationship. The solid white molecules are the intermediate factors which take part in activation of NFκB and were also observed to be upregulated.

The RT^2^ Profiler Array data was further analyzed using the Ingenuity Pathway Analysis to decipher the role of the differentially expressed genes in the cellular response to IAV infection. The analysis suggests that the knockdown of NLRC5 during IAV infection enhances the activation of the NFκB signaling pathway. In particular, seven genes that were upregulated in the absence of NLRC5 during infection were identified by this analysis as playing a direct or indirect role in the upregulation of the NFκB signaling pathway (Figure 7). For example, the increase in IL-2 directly upregulates NFκB signaling, while the enhancement of CASP-1 or TLR5 can indirectly upregulate the pathway through IL-6. While there are limitations involved in obtaining monoclonal antibodies for chicken NFκB proteins, further studies are required to provide concrete phosphorylation evidence to determine the effect of NLRC5 on NFκB signaling pathway.

## Discussion

The role of NLR family members as innate immune sensors and pathogen recognition receptors has started to surface in last few years. In particular, NLRC5 is biologically conserved in the immune tissues as well as in lung and intestines, where the host often has initial contact with pathogens (Li et al., 2014), suggesting a role in innate immunity. Further, NLRC5 is highly expressed in monocyte/macrophage cell lineages (Davis et al., 2011), which are central drivers of the host innate immune response and have a well-defined role during invasion by viral pathogens. It is also known that NLRC5 is activated during several viral infections in different cell types including Raus sarcoma virus-infected A549 cells (Guo et al., 2015), cytomegalovirus-infected HFF cells (Kuenzel et al., 2010) and Sendai virus-infected HeLa cells (Neerincx et al., 2010). To date, there have been few reports on the role of NLRC5 in IAV infection, for example it has been shown that NLRC5 is upregulated in HPAIV infected chicken by 2.9-folds (Ranaware et al., 2016). Most previous NLRC5 work has been limited to human and mouse cell-based infection systems (Benko et al., 2010; Cui et al., 2010; Davis et al., 2011; Ranjan et al., 2015). Despite this, currently available data across hosts and stimuli suggest that NLRC5 can act as both positive and negative regulator of the inflammatory immune response. In the current study, we observed a number of effects on IAV replication and host immune function in chicken macrophage, which highlight NLRC5 as a cellular pro-viral factor and moderator of cytokine expression.

This study provides strong evidence that NLRC5 is acting as a pro-viral factor in chicken macrophages during AIV infection. We first observed that NLRC5 is strongly upregulated after infection in both chicken macrophages and chicken primary lung cells (Figures 1, 2), demonstrating that AIV infection can effectively upregulate expression in various cells. This finding is in line with previous data in human cell lines showing that NLRC5 is induced following viral infection, however, the degree to which it is cell type-dependent, as we have seen here. For example, ~6-fold upregulation of NLRC5 mRNA and protein was observed in HeLa cells upon infection with Sendai virus, whereas in THP1 cells <2-fold upregulation was seen (Neerincx et al., 2010).

In addition, we observed a strong positive correlation between the upregulation of NLRC5 and increasing AIV titers, a phenomenon also observed in human cell lines (Guo et al., 2015), suggesting that NLRC5 activation leads to a more productive infection across species. Moreover, we did not observe an increase in virus replication over time in the A549 cell line where NLRC5 was not upregulated (data not shown). This was confirmed by knockdown experiments where, in the presence of decreased NLRC5, we observed decreased intracellular viral RNA and extracellular AIV (Figure 4).

NLRC5 also has the potential to serve as a pro-viral factor during AIV infection by modulating the immune response. NLRC5 has been implicated in the regulation of IFN-dependent gene transcription where its knockdown impaired the release of IP10, IFN-β and CCL5 and diminished secretion of IFN-α (Neerincx et al., 2010) suggesting NLRC5 can enhance the pro-inflammatory response. However, in addition to its role as a positive regulator of antiviral cytokines, it is also reported as a negative regulator of NFκB (Benko et al., 2010) which is a hallmark for most virus infections and gatekeeper to several key actors in the innate immune response (Santoro et al., 2003; Zhao et al., 2015). While AIV infection in the wild-type macrophage suppresses the expression of NFκB pathway modulators, this suppression was reversed when NLRC5 expression was decreased (Figure S2). The RT^2^ profiler array analysis of the change in innate immune gene expression in AIV-infected NLRC5 KD revealed activation of the NFκB pathway based on the upregulation of several key pro-inflammatory mediators including IL-2, IL-6, CCL5, IL-5, STAT3, and IFN-ν (Figures 6, 7). This observation indicates a possibility of NLRC5 being a NFκB inhibitor in chicken macrophages. Further protein-based experiments are warranted to confirm this observation. This is in line with previous studies in the mouse model which have consistently shown activation of the NFκB pathway in the absence or suppression of NLRC5 expression. Bone marrow-derived dendritic cells from NLRC5^−/−^ mice showed activation of NFκB and NFκB-dependent cytokine IL-6 after IAV infection (Lupfer et al., 2017) and shRNA-mediated knockdown of NLRC5 murine macrophages have shown induction of inflammatory cytokines such as IL-6, TNFα, and IL-1β (Benko et al., 2010). As in murine models, our data suggests a possibility that the upregulation of NLRC5 in chicken macrophages dampens the NFκB signaling pathway which could dampen the secretion of the antiviral cytokines. These results indicate a possibility that, through modulation of the NFκB pathway, NLRC5 expression results in the establishment of a pro-viral state in the cells, leading to an increase in virus replication. Further protein-based experiments are warranted to fully uncover the mechanistic details of this observation.

It has been shown that NLRC5 competes with NEMO for binding to IKKa/IKKb in 293T cells and blocks IKK phosphorylation, thus inhibiting NFκB activation (Cui et al., 2010). While such mechanistic insights are yet to be confirmed in chickens, our data suggest there may be a similar protein-protein interaction taking place, leading to inhibition of the NFκB signaling pathway. Viruses have evolved strategies to inhibit NFκB activation by interfering with most steps of NFκB signaling pathway (Mulhern et al., 2009).

Antiviral drugs face the risk of development of resistance by newly emerging virus strains. There have been reports of IAV strains which were found to be resistant to compounds which target viral proteins M2 and NA. As an alternate strategy, reports have suggested targeting host proteins which facilitate IAV replication (Shaw, 2011). In recent years, several host-protein targets have been identified as alternative or supplementary approaches to the existing antiviral strategies. NLRC5 maybe an option as a potential therapeutic target, the blocking of which can amplify the NFκB expression and help limit IAV replication. However, detailed studies are required to understand the tissue and cell-type specific role of various isoforms of NLRC5 as indicated earlier (Ranjan et al., 2015) in regulation of NFκB pathway.

In addition to enhancing activation of NFκB pathway, IAV infection in NLRC5 KD cells resulted in a significant decrease in the expression of IL-1β and IL-18 (Figure 6), the two key cytokines involved in inflammasome formation. Recent reports have highlighted the importance of the inflammasome in antiviral defense (Allen et al., 2009; Ichinohe et al., 2009; Thomas et al., 2009). It is currently thought that NLRC5 acts cooperatively with NLRP3 to activate the inflammasome-dependent secretion of IL-1β and IL-18 in response to stimuli (Davis et al., 2011; Yao et al., 2012; Pontigo et al., 2016). Further, overexpression of NLRC5 was shown to upregulate IL-1β in human hepatic stellate cells (Xu et al., 2015). While it is not clear whether inflammasome activation also takes place in chickens, our data raises a strong possibility that chicken NLRC5 could be involved in the activation of inflammasome or a related response in chicken cells.

In summary, our findings strongly suggest that NLRC5 is a negative regulator of NFκB pathway and a pro-viral factor of IAV infection in chicken, possibly involved in activation of inflammasome complexes. Further studies are warranted to identify viral factors responsible for upregulation of NLRC5 and define protein-protein interactions in the chicken which may lead to direct regulation of virus production and effect NFκB pathway regulators.

## Data Availability Statement

The datasets generated for this study can be found in the Microarray datasets are available on the gene expression omnibus (GEO) site under accession number GSE33389.

## Author Contributions

SK conceived the study. SC carried out most of the experiments. RN, SK, and SC carried out the BSL-3 infection studies. LL, GB, and IB helped in the data analysis. SK and BJ contributed to study design and supervision. SK, SC, RN, and JR-B interpreted the data and drafted the manuscript. All authors have read and approved the manuscript.

## Conflict of Interest

The authors declare that the research was conducted in the absence of any commercial or financial relationships that could be construed as a potential conflict of interest.
