# Supplementary material for: NLRC5 Serves as a Pro-viral Factor During Influenza Virus Infection in Chicken Macrophages
Source: Front Cell Infect Microbiol. 2020 May 19;10:230. doi: 10.3389/fcimb.2020.00230 (PMC7248199; doi:10.3389/fcimb.2020.00230)
Supplement: Supplementary file 1 [file Data_Sheet_1.docx]

Supplementary Material

## Supplementary Figures

**Figure S1. NLRC5 mRNA is increased in H5N2-infected CEF cells.**

**Figure S2. Heatmap showing complete data set for the RT2 Profiler Assay.**
